# Oseltamivir-Resistant Influenza A Pandemic (H1N1) 2009 Virus, Hong Kong, China

**DOI:** 10.3201/eid1512.091057

**Published:** 2009-12

**Authors:** Honglin Chen, Chung Lam Cheung, Hung Tai, Pengxi Zhao, Jasper F.W. Chan, Vincent C.C. Cheng, Kwok-Hung Chan, Kwok-Yung Yuen

**Affiliations:** The University of Hong Kong, Hong Kong Special Administrative Region, People’s Republic of China

**Keywords:** Oseltamivir resistance, quasi-species, pandemic (H1N1) 2009, influenza, viruses, China, expedited, dispatch

## Abstract

Resistance to oseltamivir was observed in influenza A pandemic (H1N1) 2009 virus isolated from an untreated person in Hong Kong, China. Investigations showed a resistant virus with the neuraminidase (NA) 274Y genotype in quasi-species from a nasopharyngeal aspirate. Monitoring for the naturally occurring NA 274Y mutation in this virus is necessary.

Emergence of influenza A pandemic (H1N1) 2009 virus, presumably from swine to humans, has spread globally since April 2009 (*1*–*3*). This emergence prompted the World Health Organization to declare a pandemic caused by this virus on June 11, 2009. Although most cases of infection are mild or asymptomatic, 1,462 fatal cases were reported to the World Health Organization as of August 6, 2009 (www.who.int/csr/don/2009_09_11/en/index.html).

Experimental evidence from animal models showed that this virus was able to replicate to high titers in the lungs of infected animals (*4*), unlike seasonal influenza viruses, which mainly infect the upper respiratory tract. Serologic studies found that antibodies induced by current seasonal influenza vaccines show little cross-reactivity to pandemic (H1N1) 2009 virus (*5*).

Therapeutic options are presently limited to 2 neuraminidase (NA) inhibitors, oseltamivir and zanamivir, because this virus has a swine-origin matrix 2 (M2) gene, which contains a mutation associated with resistance to M2 ion channel blockers amantadine and rimantadine. Although oseltamivir has been widely used in persons infected with pandemic (H1N1) 2009 virus, resistance was not observed until recently. Three unrelated cases of resistance to oseltamivir were observed in Denmark, Japan, and Hong Kong (www.who.int/csr/disease/swineflu/notes /h1n1_antiviral_resistance_20090708/en/index.html).

Emergence of resistance to oseltamivir by seasonal influenza A virus (H1N1) was detected in Norway in 2007. This virus has evolved into the dominant influenza A virus (H1N1) in humans (*6*). This finding raises strong concerns that the 274Y resistant mutation in pandemic (H1N1) 2009 virus might circulate and become dominant. We report virologic investigation of the emergence of oseltamivir resistance in this virus in a patient from Hong Kong.

## The Study

A 16-year-old previously healthy girl had a fever at the Hong Kong International Airport after her arrival from San Francisco, California, USA, on June 11, 2009. Physical examination showed a temperature of 38.3°C, a blood pressure of 117/66 mm Hg, a pulse rate of 94 beats/min, and an oxygen saturation of 99% at room air. Results of a complete blood count and liver and renal function tests were normal. She had a leukocyte count of 4.69 × 10^9^ cells/L, an absolute neutrophil count of 2.36 × 10^9^ cells/L, and a lymphocyte count of 1.74 × 10^9^ cells/L. Findings on her chest radiograph were normal.

A nasopharyngeal aspirate (NPA) was positive for influenza A virus (H1N1) nucleoprotein by immunofluorescence. NPA specimens on days 1 and 5 were positive for influenza A virus (H1N1) M gene and swine-specific specific H1 gene by reverse transcription–PCR (RT-PCR). Samples obtained on days 6–8 were negative. Serum and midstream urine specimens obtained on day 2 were negative for influenza A virus (H1N1) M gene by RT-PCR.

The patient refused antiviral therapy with oseltamivir because of fear of its potential side effects. She was then offered symptomatic treatment. Her clinical condition gradually improved and she was discharged on day 8 uneventfully.

Influenza A pandemic (H1N1) 2009 virus was cultured from NPA. Subsequent drug susceptibility testing showed that this isolate was resistant to oseltamivir (50% inhibitory concentration 197.5 nM), but susceptible to zanamivir, as determined by enzymatic assay (Table).

**Table:** Quasi-species of 274H and 274 Y pandemic (H1N1) 2009 virus from NPA samples and subsequent virus isolate A/Hong Kong/2369/2009 from MDCK cells conferring resistance to oseltamivir, Hong Kong, China*

Sample	274H, no. samples positive/no. tested (%)	274Y, no. samples positive/no. tested (%)	IC_50_ for oseltamivir, nM	IC_50_ for zanamivir, nM
NPA	45/95 (47.37)	50/95 (52.63)	ND	ND
MDCK cell culture	2/96 (2.08)	94/96 (97.92)	197.5	0.8

*50% inhibitory concentrations (IC_50s_) for oseltamivir and zanamivir were determined by using NA-Star influenza neuraminidase (NA) inhibitor resistance detection kits (Applied Biosystems, Foster City, CA, USA) according to the manufacturer’s instructions. Ratios of 274H and 274Y were evaluated by cloning neuraminidase (NA) gene PCR products from nasopharyngeal aspirate (NPA) samples or MDCK cell–cultured virus into the TA vector (Invitrogen, Carlsbad, CA, USA) and sequencing clones containing the NA gene. ND, not done.

To confirm whether the virus contained mutations associated with resistance to NA inhibitors, NA sequences from the day 1 NPA specimen and an MDCK cell isolate were examined. Viral RNA was extracted from NPA and MDCK cell supernatants by using reported procedures (*7*). RT-PCR was performed by using primers spanning position 274 of the NA gene (forward: 5′-ACACAAGAGTCTGAATGTGCATGT-3′; reverse: 5′-GTCTCCGAAAATCCCACTGCATAT-3′). Direct sequencing of PCR products was performed by using a BigDye Terminator v3.1 cycle sequencing reaction kit on an ABI PRISM 3730 DNA analyzer (Applied Biosystems, Foster City, CA, USA).

Sequences indicated that the NA genes in the NPA and MDCK cell virus isolates contained an H→Y mutation at the NA 274 (H3 numbering, 275 in H1 numbering) residue (GenBank accession no. GQ351316). No other NA mutations known to be associated with oseltamivir resistance were observed. Further examination of sequences showed mixed populations (T/C) in the NA gene from the NPA specimen (Figure, panel A).

**Figure:**
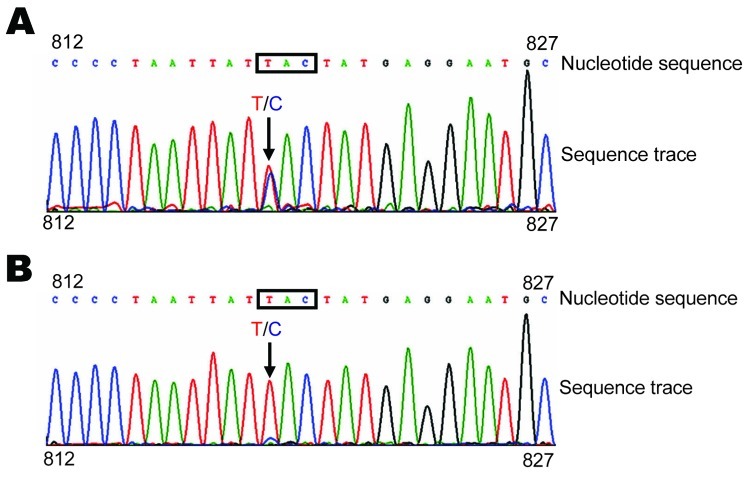
Neuraminidase (NA) 274Y (H3 subtype numbering) gene mutation in influenza A pandemic (H1N1) 2009 virus A/Hong Kong/2369/2009 isolated from a patient who arrived in Hong Kong, China, from San Francisco, California, USA, on June 11, 2009. A) NA sequence of virus amplified by reverse transcription–PCR and sequenced directly from a day 1 specimen of a nasopharyngeal aspirate from the patient. B) NA sequence of virus grown in MDCK cells. Nucleotide sequence represents identification of nucleotides by the sequencing machine, and the sequence trace represents the signal (peak) of each nucleotide in the sequencing reaction. Nucleotide coordinates (812 and 827) refer to the NA gene sequence of pandemic (H1N1) 2009 virus. Residue 274Y encoded by the 3-nucleotide codon is indicated in boxes and the nucleotide substitution (C→T for amino acid change H→Y) is indicated by arrows Colors of curves match those of specific nucleotides.

Estimation of 274H and 274Y populations in the NPA specimen was performed by cloning and sequencing PCR products. The NPA specimen contained approximately equal proportions of 274Y and 274H (52.63% and 47.37%, respectively). Examination of sequences from the MDCK cell isolate showed predominantly the 274Y type, although a minor 274H peak was also observed (Figure, panel B). Cloning and sequencing of PCR products from the MDCK virus isolate showed that 97.92% of the NA genes were 274Y, which suggests that the 274Y population overtook the 274H population during MDCK cell culture.

## Conclusions

Resistance to NA inhibitors among seasonal strains of human influenza viruses (A/H1N1, A/H3N2, and B) has been rare until recently. Development of resistance after oseltamivir treatment has occurred in 0.33%–5.5% of treated patients (*8*). Oseltamivir resistance associated with the NA 274Y genotype was also observed in human infections with avian influenza A virus (H5N1) (*9*,*10*). Low levels of 274Y quasi-species in avian influenza A viruses (H5N1) from avian hosts has been reported (*11*). Oseltamivir-resistant human influenza A viruses (H3N2 and H1N1) have been found to replicate less efficiently than oseltamivir-susceptible strains in cell culture and animal models (*12*–*14*). However, the NA 274Y resistant mutant in highly pathogenic avian influenza A virus (H5N1) retained the high pathogenicity of wild-type virus in mammalian species (*15*).

In 2007, an NA H274Y oseltamivir-resistant variant of seasonal influenza A virus (H1N1) was detected in Norway (*6*). This virus has now become the dominant virus population globally, overtaking oseltamivir-susceptible influenza A virus (H1N1). The molecular basis for the 274Y variant in seasonal influenza A virus (H1N1) virus and the mechanism by which this resistant variant became the dominant population remain unknown.

Lack of general immunity to pandemic (H1N1) 2009 virus in the human population, combined with the inherent adamantane resistance of the virus, indicates that NA inhibitors constitute the primary treatment regimen for susceptible patient groups and those in whom severe diseases develop during the current pandemic. There is great concern that an oseltamivir-resistant variant of pandemic (H1N1) 2009 virus may emerge and circulate in a manner similar to oseltamivir-resistant seasonal influenza A virus (H1N1).

The patient in this study was not treated with oseltamivir. Therefore it is unlikely that the 274Y mutation was drug-induced. Detection of mixed populations of 274Y and 274H in the NPA specimen before antiviral treatment suggests that the mutation occurs naturally, either before or during infection. Although no experimental data exist that show the growth properties of this resistant variant, examination of the quasi-species population in the cell culture–propagated virus isolate showed that the 274Y variant has become the dominant population. This finding implies that the 274Y mutation does not compromise replication of pandemic (H1N1) 2009 virus in vitro.

Quarantine procedures adopted by the Hong Kong Special Administrative Region in China during the early containment phase might have limited transmission of this variant virus. Knowledge of this virus is still limited, and characterization of transmission properties of this resistant variant in in vitro and in vivo models is needed. Moreover, pandemic (H1N1) 2009 virus should be closely monitored for emergence of resistant variants.
